# Tooth-on-a-chip to engineer early dental epithelial-mesenchymal interaction

**DOI:** 10.1016/j.mtbio.2025.102451

**Published:** 2025-10-23

**Authors:** C. Huang, F. Sanaei, W. Zhang, P.C. Yelick, W. Ji, F. Yang, X.F. Walboomers

**Affiliations:** a Department of Dentistry – Regenerative Biomaterials, Research Institute for Medical Innovation, Radboud University Medical Center, Philips van Leijdenlaan 25, 6525 EX, Nijmegen, the Netherlands; b State Key Laboratory of Oral & Maxillofacial Reconstruction and Regeneration, Key Laboratory of Oral Biomedicine Ministry of Education, Hubei Key Laboratory of Stomatology, School & Hospital of Stomatology, Wuhan University, China; c Department of Orthodontics, Division of Craniofacial and Molecular Genetics, Tufts University School of Dental Medicine, Boston, MA, 02111, USA; d Department of Implantology, School & Hospital of Stomatology, Wuhan University, Wuhan, China

**Keywords:** Tooth regeneration, Organ-on-a-chip, Ameloblast, Odontoblast, Mineralized tissue

## Abstract

Tooth enamel—the highly mineralized outer layer shielding teeth from mechanical and chemical wear—is produced during development by specialized dental epithelial (DE) cells called ameloblasts. Functional enamel is challenging to regenerate because the enamel-forming cells—DE-derived ameloblasts—disappear even before tooth eruption. To achieve regeneration, DE and dental mesenchymal (DM) need to interact. Current in-vitro models encompass only limited DE–DM interaction as they offer little control over the interface geometry and usually force both lineages to share a single, sub-optimal medium, leading to variable outcomes. Hence, herein we developed a micro-engineered “tooth-on-a-chip” that shapes the DE–DM interface while independently perfusing lineage-specific media. A three-channel polydimethylsiloxane (PDMS) device, fabricated by rapid 3-D printing and soft lithography, traps DM cells in a fibrin hydrogel between two fluid channels. Within 3 days the DM cells condensed into a papilla-like core and secreted collagen, creating a signaling niche for sequential seeding of DE cells. Quantitative PCR (qPCR) of DM cells showed progressive up-regulation of odontogenic markers, while immunofluorescence confirmed ameloblast differentiation of the DE layer through AMELX expression. Alizarin Red staining, calcium assays, SEM confirmed progressive, spatially confined mineralization at the engineered interface. Although this platform now is in its proof-of-concept stage, it provides the first controllable, dual-medium microenvironment that recapitulates early odontogenesis, amelogenesis, and mineral deposition in a single device, offering a versatile tool for dissecting tooth morphogenesis and accelerating true enamel-regeneration strategies.

## Highlights

• Separate medium channels supply lineage-specific media to DE and DM cells.
• DM cells organize into a papilla-like structure that shapes the DE–DM interface and provides developmental cues.
• The chip supports organized odontogenesis, amelogenesis, and mineralization for prolonged culture periods up to 25 days.

## 1 Introduction

Tooth loss affects millions of people daily, impairing chewing, causing pain, and diminishing appearance-related confidence. Innovatively diverging from traditional synthetic material restoration, a significant leap in modern dentistry is the creation of bioengineered tissues that mimic natural tooth in both form and function [1].

To construct functional dental tissue *in vitro*, researchers have focused on an initial functional structure of the *in vivo* dental placode, dental epithelial (DE) and dental mesenchymal (DM) interface [2]. In natural tooth development, reciprocal DE–DM signaling drives DE cells to form enamel-secreting ameloblasts and DM cells to form dentin-producing odontoblasts. Because ameloblasts are lost before eruption, mature enamel is incapable of self-repair, making an accurate *in vitro* recreation of the DE–DM interface critical for regenerative strategies.

Over the last decades, several *in vitro* DE-DM models have been developed, including compartmentalizing DE and DM cells in collagen [3], bilayer cell sheets [4], in self-assembling organoids [[5], [6], [7]], and cell-laden (hydrogel-)scaffolds [[8], [9], [10]]. These models have provided insights into tooth developmental processes, notably demonstrating odontoblast and ameloblast differentiation *in vitro*. Yet they still yield variable outcomes *in vivo* and lack control over interface geometry, tooth number, and orientation [11,12]. Despite this progress, all current systems share a critical limitation: none actively controls the size or curvature of the DE–DM interface. Interface curvature and surface area set morphogen gradients (e.g. BMP4, SHH, Wnt) and mechanical cues that regulate cusp numbers, patterning, and odontoblast condensation [13,14]. Equally important, most existing platforms maintain both lineages in a single, compromise medium, rather than providing each cell type with conditions tailored to its distinct nutritional and signaling needs.

**Fig. 1:**
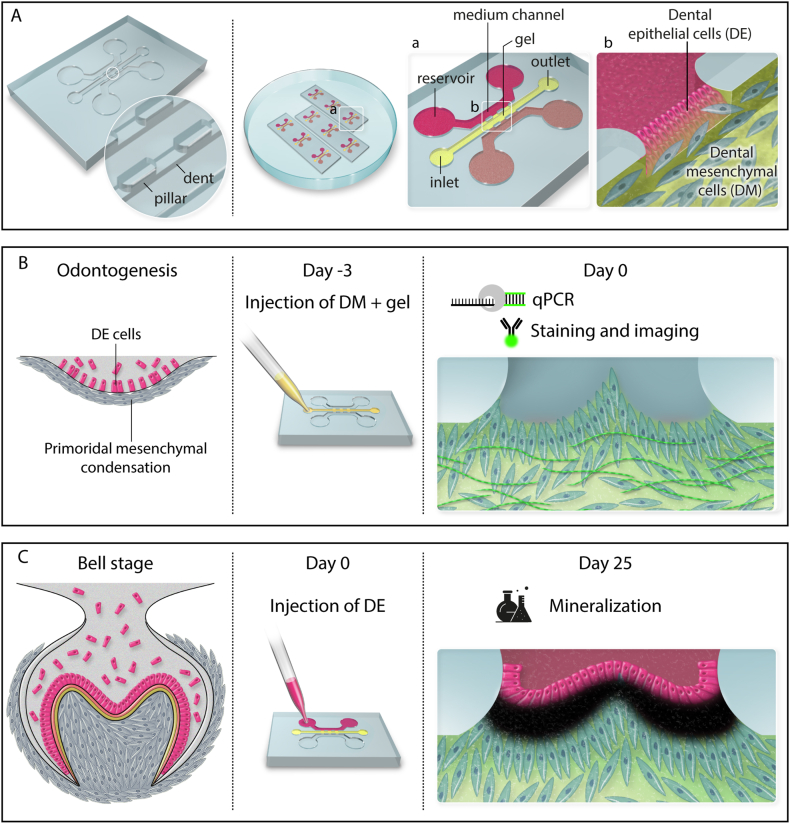
**Schematic overview of tooth-on-a-chip model.** A) Structural overview of the tooth-on-a-chip platform. The left panel illustrates the chip structure, which contains 3 parallel channels and two rows pillars. Several chips can be cultured simultaneously. A higher magnification view (a) shows the side channels with reservoirs filled with DE- and DM-specific media, respectively (light/dark purple), while the central channel contains a DM-gel mixture (yellow). At even higher magnification (b) the DE-DM cell interface is shown between the pillars, where DE cells adhere to the DM-gel matrix. B) Mimicking mesenchymal condensation and osteogenesis. Left; In natural tooth development, mesenchymal cell condensation is characterized by locally increased DM density beneath the epithelial layer, driving DM cell differentiation. Middle: DM cells were mixed with gel and cultured in chip for 3 days. Right: the perpendicular self-aligned DM cells expressed odontogenic transcription factors and produced collagen I. C) Tooth-on-a-chip culture mimicking the bell stage. Left: in natural tooth development the dental organ is bell-shaped during this stage, with organized ameloblast, enamel, dentin, and odontoblasts in a layered structure. Middle: DE cells were introduced from the side channels to interact with constructed DM on chip. Right: the system replicated the bell shape and the sandwich-like structure of the enamel-dentin complex with abundant evidence of mineralization specifically located between ameloblasts and odontoblasts.

**Fig. 2:**
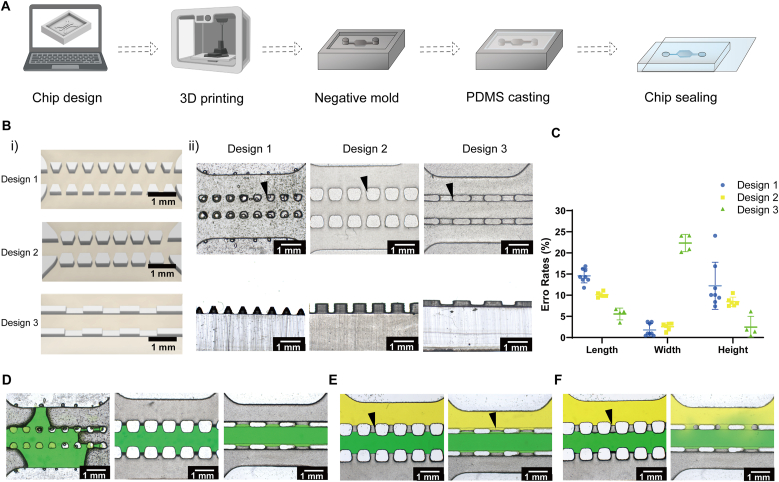
**Fabrication method and functional validation of different chip designs.** A) Stepwise chip fabrication process. B) Computer-aided designs (i) and light microscopic images (ii) of fabricated chips from top and longitudinal views for Design 1 with trapezoidal pillars, Design 2 with larger hexagonal pillars, and Design 3 with an additional “dent” structure in between each pillar. Rounded edges are indicated with black arrows. C) Quantitative histograms of fabrication accuracy of different-designed chips in width, length, and height. D) Light images (top view) of the chips with water (green) injection into the central channel. Note the failure of design 1 with evident sideways leaking. E) Light images (top view) of the chips with water injection into the central (green) and side (yellow) channels simultaneously. Bubbles are indicated with black arrows F) same as E) but after a 10-min incubation period. Note that bubbles gradually disappear only in design 3, which was selected as suitable for further experimentation.

**Fig. 3:**
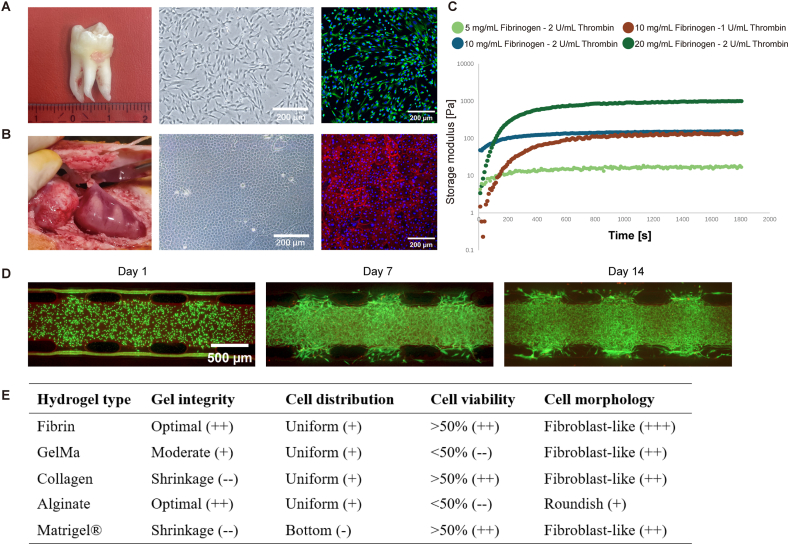
**Cell characterization, gel properties, and gel cytocompatibility in 3D culture**. A) Left: a representative human third molar from which DM cells were harvested; Middle: light micrograph of DM cells; and Right: positive immunostaining for vimentin (green). B) Left: tooth bud retrieval; Middle: light micrograph of cultured DE cells; and Right: positive immunostaining for E-cadherin (red). C) Dynamic storage moduli during gelation of fibrin gels with varying fibrinogen and thrombin concentrations, as thrombin activated fibrinogen to form a crosslinked fibrin gel. D) Live-dead staining of 3D cultured DM cells in 10 mg/mL fibrinogen-2 U/mL thrombin on days 1, 7, 14. Note the abundance of live cells (green), whereas dead cells were almost absent (red). E) Table summarizing system stability, homogeneity, cell viability, and morphological changes of various hydrogels loaded with DM cells.

Here, we present a tooth-on-a-chip platform that biomimetically replicates the DE-DM interface, addressing the lack of reliable *in vitro* models for early-stage tooth development. To achieve this, we specifically sought to: (1) define an optimal design for DE-DM interface construction, allowing for separate media flow to both cell types (Fig. 1A); (2) replicate the shaped dental papilla of embryological bell-stage teeth, establishing a dynamic signaling niche to fster differentiation of DE cells (Fig. 1B); (3) maintain viable tooth-on-a-chip constructs for 25 days to observe developmental processes; and (4) validate the progression of natural tooth development, including odontogenesis, amelogenesis, and mineralization stages (Fig. 1C). This comprehensive approach allowed for the development of a robust, functionally representative “tooth-on-a-chip" facilitating future studies into tooth regeneration.

## 2 Materials and methods

### 2.1 Chip design, fabrication, and selection

Three distinct chips were designed in Autodesk® (San Rafael, CA) each with three parallel channels separated by two rows of pillars. The central channel was 0.7 mm, and side channels were 0.86 mm in width. The designs varied in pillar shape and size. Design 1 featured commonly used trapezoidal pillars [15,16] of 0.13 mm^2^ × 0.4 mm. Smaller pillars can hinder bonding the silicon chip to a cover glass, therefore Design 2 incorporated hexagonal pillars with larger surface area (0.4 mm^2^), ensuring effective bonding and consistent liquid pinning between pillars. However, increasing the interpillar distance would enhance the available interface area. Therefore, Design 3 (0.3 mm^2^ bonding) incorporated larger inter-pillar distance (0.8 mm) with a “dent” structure in between, to further optimize interface formation.

Stereolithography-based (SLA) 3D-printing (Formlabs, Somerville, MA) was used to fabricate negative template molds. Liquid Sylgard 184 and curing agent (10:1; Dow Corning, Midland, MI) were poured onto the molds, cured (65 °C, 2h), and peeled off, thus replicating the desired architectures (Fig. 2A).

Inlets of the central and channels and reservoirs at the end of side channels were created with 1 and 4 mm biopsy punches. PDMS and cover slips were treated with air plasma (300mTorr, 1min; Harrick, New York, NY) before compressing manually. The so-formed chips were transferred to 65 °C for at least 2h to complete bonding.

The 3 distinctive designs were then tested for the following parameters. (1) Fidelity: PDMS pillar structures were measured (l × w × h) using calibrated digital microscopy, and accuracy was calculated by comparing dimensions with the computer-aided designs. (2) Hydrogel caging: To assess capacity of chips to retain gel in the central channel, green-dyed water was injected into the central channel and the distribution was evaluated by microscopy. Water was chosen purposely as being less viscous compared to gel (i.e. “if it works for water, it will also work for any gel”). (3) Medium transport capacity: Yellow-colored water was subsequently introduced into the side channels after filling the central channel. Following (10min, 37 °C), the presence of air bubbles between pillars was investigated, as these could impede efficient medium flow.

### 2.2 Primary cell retrieval and culture

Porcine dental epithelial (DE) cells and primary human dental mesenchymal (DM) cells were extracted by enzymatic digestion [17]. DE cells without further passaging were cultured in LHC-8 medium (Gibco, Waltham, MA) with 10 vol% fetal bovine serum (FBS) and 1 vol% penicillin-streptomycin (P/S). DE cells were characterized in brightfield imaging for correct cuboidal phenotype, and by immunostaining with rabbit anti-E-cadherin (1:40; Antibodies Online, Aachen, Germany). DE cells were cultured on the chip in differentiation medium containing dexamethasone (10^−8^ M), β-glycerophosphate (10 mM), and Vitamin C (50 μg/mL).

DM cells of passage 3–6 were characterized by immunostaining with mouse anti-Vimentin (1:100; Invitrogen, Eugene, OR), in α-MEM medium (Gibco) with 10 vol% FBS and 1 vol% P/S. DM cells were pre-differentiated for 2 days in differentiation medium with the same dexamethasone, glycerophosphate and Vitamin C differentiation components, prior to loading into chips.

### 2.3 Mechanical testing of fibrin

Fibrin was formed via thrombin-mediated polymerization of plasma fibrinogen. Rheology was performed to optimize the gel composition for (1) shorter gelation time to avoid cell sedimentation and (2) stiffness resembling native pulp [18,19]. The storage modulus (G′) was measured to assess hydrogel stiffness, and dynamic changes in stiffness during gelation.

Human fibrinogen solutions (22.2 mg/mL and 11.1 mg/mL) were prepared in saline/thrombin solutions (40U/mL and 20U/mL) (Sigma, St. Louis, MO). Fibrin solutions were mixed by fibrinogen, thrombin, and calcium-chloride (50 mM) solutions at 18:1:1 (v/v) ratio. After mixing, liquid fibrin was injected into a rheometer (TA, Newcastle, DE), for sweep-testing (30min, 1 % strain, frequency 1 rad/s, 37 °C).

### 2.4 Tooth-on-a-chip assembly

Pre-differentiated DM cells were injected into the chip at 2 × 10^6^ cells/mL in 10 mg/mL fibrin. Following a 15-min incubation period to allow gel polymerization and cell embedding, culture medium was introduced into the side channels. To prevent fibrin degradation by plasmin, aminocaproic acid (ACA; Sigma, 40 μg/mL) was added to the culture medium. After 3 days of culture, 5 × 10^5^ cells/mL DM cell suspension was loaded from the upper channel to form a “feeder layer” onto the gel surface, which reduced electrostatic repulsion between negatively charged DE cells and fibrin and thereby enabled DE adhesion. On day 4, DE cells (5 × 10^5^ cells/mL) were introduced into the same channel. DM differentiation medium was supplied to the lower, and DE differentiation medium to the upper channel. Culture medium was manually refreshed every two days.

### 2.5 Fluorescent staining and imaging on chip

Besides fibrin also other hydrogels were tested, including 5 % gelatin methacryloyl (GelMA) [20], 3 mg/mL collagen (Dow Corning, MI), 2 % alginate [21], and 8 mg/mL Matrigel (Dow Corning). Evaluation focused on system stability, cell morphology, and viability on days 1, 7, and 14 (Live/Dead Kit, Invitrogen).

Cell alignment was analyzed on days 3, 5, and 7 by staining with phalloidin-Alexa Fluor 488 (1:50, Invitrogen). The alignment angle distribution was simulated with the OrientationJ plugin in ImageJ (NIH, Bethesda, MD) normalized by Gaussian nonlinear regression. The full width at half maximum (FWHM) of the angle peak was calculated to quantify the spread of the alignment angles. Collagen distribution was visualized on day 3 with Oregon Green (CNA35-OG488, Gibco).

After confirming cross-species reactivity, immunostaining was performed on day 21 (rabbit anti-Amelogenin 1:100, Millipore, Burlington, MA; rabbit anti-Ameloblastin 1:100, Proteintech, Rosemont, IL, USA) overnight at 4 °C, followed by incubation with Alexa Fluor 568-conjugated goat anti-rabbit IgG (1h, 1:200, Invitrogen). Nuclei were counterstained with DAPI (1h, 1:100, Invitrogen).

Washing and staining solutions were introduced through side channels to ensure penetration into the central cell-gel mixture. Confocal microscopy (Zeiss, Gottingen, Germany) was used for 3D reconstruction, with maximum intensity projections generated in ImageJ.

### 2.6 Real-time qPCR analysis

qPCR was conducted on mesenchymal condensation genes (*PAX9, BMP4, MSX1*) and the early odontogenetic genes (*MMP9, ALPL, RUNX2 and COL1A1*). Briefly, total RNA was isolated from DM cells in fibrin in Trizol and synthesized into cDNA. RT-PCR reactions (4titude, Leiden, the Netherlands) were performed with SYBR Green system (Roche, Mannheim, Germany). qPCR was performed using a LightCycler 480 system (Roche) with the following cycling parameters: an initial polymerase activation at 95 °C for 10 min, followed by 45 amplification cycles comprising denaturation at 95 °C for 10 s and annealing/extension at 56 °C for 5 s. A melt curve analysis was performed from 56 °C to 96 °C in 0.6 °C increments [22]. Relative mRNA expression levels were calculated using the 2^−ΔΔCt method, with normalization to the housekeeping gene *HPRT*. Table 1 shows sequences of all primers used.

**Table 1:** Primer sequences for target genes analyzed by qRT-PCR.

Target Gene	Primers Nucleotide sequences 5′-3′
*HPRT*	FW GCTGACCTGCTGGATTACAT REV CTTGCGACCTTGACCATCT
*COL1A1*	FW TCCGGCTCCTGCTCCTCTTA REV GGCCAGTGTCTCCCTTG
*ALPL*	FW AGGGACATTGACGTGATCAT REV CCTGGCTCGAAGAGACC
*MSX1*	FW TCAAGCTGCCAGAAGATGC REV TCGGCGATGGACAGGTACT
*RUNX2*	FW ATGCTTCATTCGCCTCAC REV ACTGCTTGCAGCCTTAAAT
*MMP9*	FW TGACAGCGACAAGAAGTG REV CGTGGCTCAGGTTCAGG
*PAX9*	FW GGTGTACTGCTCGGAGCAAT REV TTGTATCGCGCCAGGATCT

### 2.7 Mineralization assay

Samples were collected on days 0, 7, 14, and 21 of differentiation. After washing, the PDMS was separated from the cover glass, and samples were incubated in HCl (200 μL, 0.6M, 2 days). Calcium content was quantified using an o-cresolphthalein colorimetric assay (Randox, WV).

### 2.8 Hematoxylin–eosin staining

Samples were collected and fixed in 4 % paraformaldehyde for 30 min, embedded in OCT, and cryosectioned at −20 °C (5 μm). Sections were air-dried, rinsed, stained with Delafield hematoxylin (Sigma-Aldrich) for 8 min, and washed in running tap water for 10 min to blue. Slides were then dehydrated through 50 %, 70 %, 80 %, and 96 % ethanol, counterstained with eosin for 2 min, further dehydrated in 90 % ethanol for 1 min and 100 % ethanol for 2 min, cleared in a 1:1 (v/v) xylene:ethanol mixture followed by two xylene baths (5 min each), and mounted with DPX. Coverslips were applied, and slides were dried overnight in a fume hood before imaging.

### 2.9 Alizarin red staining

Samples on day 25 were fixed in 70 % ethanol, washed, and stained in alizarin red (40 mM, pH = 4.2, Sigma) introduced from the side channels. Whole chips were extensively washed in water for 2 days to remove excess staining.

### 2.10 Scanning Electron microscopy

On day 21, samples were fixed overnight in 4 % neutral-buffered formalin, then rinsed twice with PBS. The PDMS lid was carefully peeled away to fully expose the 3D constructions. Samples were freeze-dried to stabilise the matrix and deposited minerals. Dried specimens were secured on aluminium stubs with carbon adhesive and sputter-coated with a 7 nm Au/Pd film. Imaging was performed on a field-emission SEM at 8 keV accelerating voltage, 0° stage tilt, 35° take-off and 90° azimuth angles under high vacuum. Elemental analysis of mineral crystals was carried out with an attached energy-dispersive X-ray spectroscopy (EDX) system (4096 channels, 123 eV Mn Kα resolution).

### 2.11 Statistical analysis

The Shapiro-Wilk test was used to assess the normality of the cell alignment, qPCR, and calcium deposition data, followed by one-way analysis of variance (ANOVA) with a post-hoc Tukey-Kramer multiple comparisons test (GraphPad, San Diego, CA). p-Values were considered significant at p < 0.05. In all cell experiments triplicates (n = 3) were used. Adjustment of fluorescent images was performed to improve the visual presentation without altering the original data integrity.

## 3 Results

### 3.1 Chip fabrication and selection

After gently removing the chips from the mold, the pillars in each of the 3 designs were evaluated from all perspectives for shape and size (Fig. 2B). Top views demonstrated varying degrees of geometric distortion across all designs, characterized by the rounding of sharp edges. Longitudinal views showed that trapezoidal pillars exhibited considerable narrowing towards the top. Although all designs achieved dimensional accuracy with error rates <30 % (Fig. 2C), Design 1 was rendered unsuitable because of apparent variability in pillar height.

Both Designs 2 and 3 successfully prevented water from leaking into side channels (Fig. 2D and E). However, entrapped air bubbles remained visible in design 2, potentially impeding medium transport from the side channel to the central channel in later cell experiments. In contrast in design 3 bubbles that initially formed shrank away over time (Fig. 2F), thus this design was selected for all subsequent experiments.

### 3.2 Hydrogel optimization and cytocompatibility in 3D culture in chip

DM cells displayed typical fibroblast morphology, and positive expression for Vimentin. DE cells exhibited a cobble-stone phenotype, and expressed E-cadherin (Fig. 3A and B).

Rheological results revealed gelation rate, time, and stiffness of the final hydrogel (Fig. 3C). Increasing the thrombin concentration from 1U/mL to 2U/mL accelerated the gelation process from 1000s to 300s but did not affect the final gel stiffness. In contrast, stiffness increased from 10Pa to 1000Pa when fibrinogen was increased from 5 mg/mL to 20 mg/mL. Therefore, 10 mg/mL fibrinogen-2U/mL thrombin was selected for all subsequent experimentation based on similarity to the biomechanical properties of native pulp tissue [18,19] and the relatively shorter gelation.

Live-dead staining determined the cytocompatibility (Fig. 3D). After injection onto the chip and gelation, nearly all DM cells remained alive on day 1, indicated by visual inspection. Additionally, the entire 3D system remained intact with abundant viable cells throughout the entire 2 week observation period.

Comparing various hydrogels (Fig. 3E) showed that Collagen and Matrigel exhibited significant shrinkage that disrupted the integrity of the constructs, making them unsuitable for long-term culture. Also, Matrigel showed most cells sinking towards the bottom of the channel. GelMA and alginate demonstrated low cell viability, and alginate showed limited cellular fibroblast-like morphology. Overall, fibrin gel met all the requirements and therefore selected for subsequent experimentation.

### 3.3 Odontogenesis on chip

**Fig. 4:**
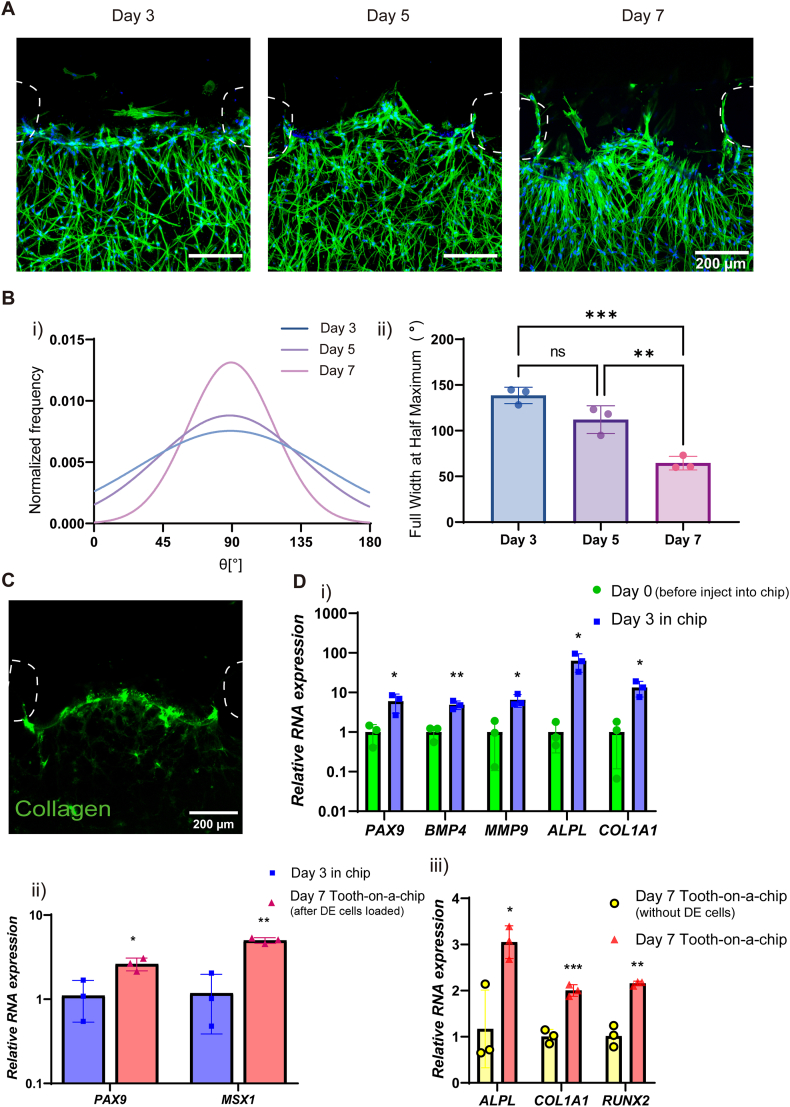
**DM cell alignment and differentiation on the chip.** A) Confocal micrographs and B) quantitative analysis of DM cell alignment between pillars on days 3–7 with fluorescent staining of nuclei (DAPI, blue) and cytoskeleton (phalloidin, green). Pillar positions are indicated with the white dotted circles. The top black area represents the side channel, while the lower region containing cells corresponds to the central channel. C) Confocal images of collagen deposition on day 3. D) Gene expression analysis of DM cells in the chip cultures. i) Relative mRNA expression of condensation markers and early odontogenic markers after 3 days of culture in differentiation medium (n = 3). ii) Relative mRNA expression of condensation markers after 7 days of co-culture with DE cells (n = 3). iii) Relative mRNA expression changes of early odontogenic markers after 14 days of culturing in the chip with or without DE cells (n = 3). Note the significant and strong upregulation for all genes.

Mesenchymal condensation is an early hallmark of odontogenesis, defining future tooth sites, initiating DE-ameloblast differentiation, and guiding crown morphogenesis. To replicate this process, DM cells underwent odontoblast differentiation before DE cell loading onto the chip. Odontoblast differentiation was assessed through measuring cell alignment, expression of early odontogenic differentiation markers, and collagen staining. DM cells reorganized perpendicularly to the openings between pillars (Fig. 4A). The main peak angle (θ) around 90° on day 3 indicated a predominant alignment of DM cells toward the gel surface. Over time, this peak became narrower and more pronounced, indicating an increased number of cells that realigned. This alignment event also was confirmed by quantitative FWHM measurements (Fig. 4B).

Moreover, fluorescent microscopy showed collagen expression at the future DE-DM interface, a key feature of amelogenesis induction Fig. 4C). All mesenchymal condensation genes and early odontogenetic genes showed significant upregulation after 3 days (Fig. 4Di). By day 7, the gene expression levels of *PAX9* and *MSX1* remained consistently elevated in the co-cultured samples (Fig. 4Dii). Compared to DM cells cultured alone within the chip, those co-cultured with DE cells exhibited significantly higher expression of early odontogenic differentiation markers on day 14 (Fig. 4Diii). This sustained increase suggests a potential inductive effect of DE cells on DM cells, indicating active early-stage signaling at the developing tissue interface.

### 3.4 Characterization of the DE-DM interface, amelogenesis and mineralization on chip

To validate the biological relevance of the chip model, we assessed amelogenesis and biomineralization of co-cultured DM-DE cells on chips. The system proved robust during extended culture times up to 25 days, without a single chip breaking, leaking or showing contamination.

**Fig. 5:**
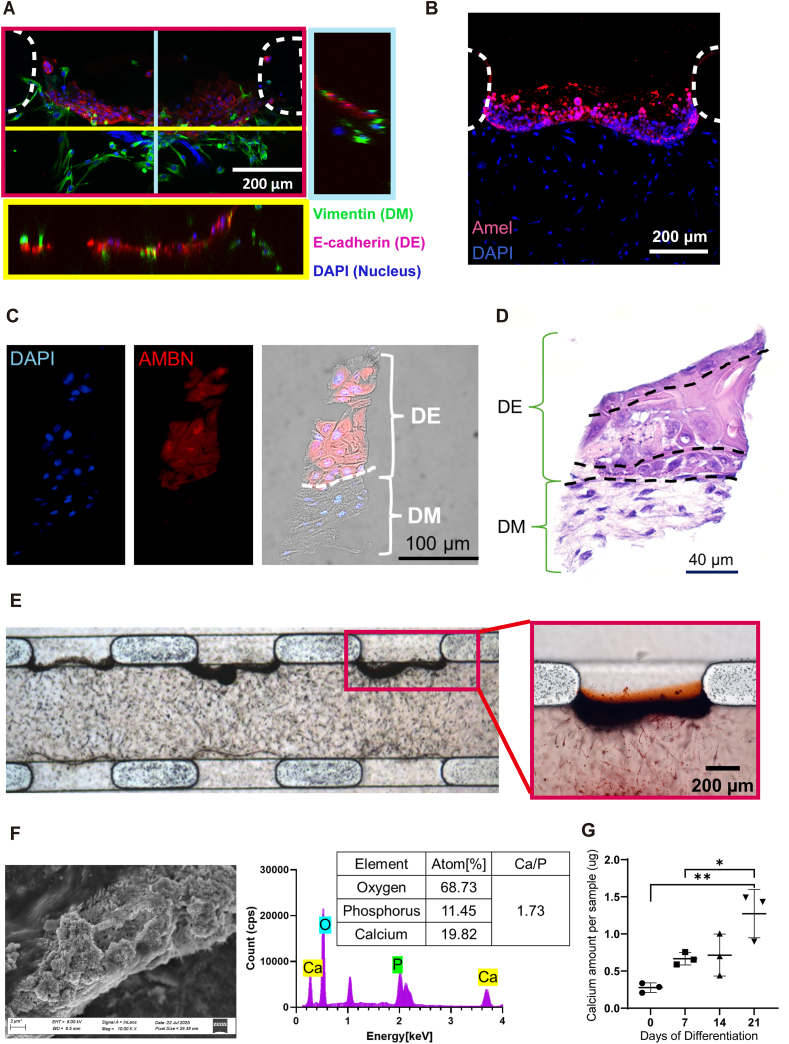
**Characterization of the DE-DM Cell Interface, Amelogenesis, and Mineralization on the tooth-on-a-chip.** A) Reconstructed confocal images on day 1, displayed in three perspectives: x-y (red box), y-z (blue box), and x-z (yellow box), with immunofluorescent staining of DM cells (green), DE cells (red), and nuclei (blue). B) Confocal micrograph on day 21 with immunofluorescent staining of nuclei (DAPI, blue) and Amelogenin (red). C) Confocal fluorescence images on day 21 showing nuclei (DAPI, blue) and Ameloblastin (red), together with a merged image overlaid with brightfield (right) to visualize cell morphology and spatial localization. D) Hematoxylin–eosin staining of day 21 samples, illustrating overall tissue organization at the DE–DM interface. E) Optical image of tooth-on-a-chip on day 11, note the dark areas of mineralization. Higher magnification of the dotted area in left panel with additional Alizarin red staining for mineralization on day 25. F)Representative field-emission SEM micrograph of the DE-DM interface on day 21 (left). The adjacent panel presents the corresponding EDX spectrum and atomic-percentage table, highlighting Ca and P peaks and a calculated Ca/P ratio. G) Quantification of mineral deposition up to day 21 (n = 3).

Visual inspection confirmed that DE cells attached to fibrin gel and established interaction with DM cells in the superficial layer. Interestingly, all interfaces adapted an invaginated morphology resembling that of the earliest bell-stage tooth development (Fig. 5A). The epithelial-ameloblast differentiation was confirmed by expression of amelogenin (Fig. 5B) and ameloblastin (Fig. 5C) specifically in the regions where DE had originally settled. H&E staining further revealed a well-organized DE–DM interface and an enamel organ–like architecture, characterized by basal polarized columnar cells, a looser suprabasal layer of polygonal/cuboidal cells, and a superficial layer of flattened cells (Fig. 5D). Brightfield imaging revealed dark dots in the central channel, particularly thickened at the DE-DM cell interface, indicating mineralized matrix deposition (Fig. 5E). This finding was confirmed by Alizarin red staining, showing strong coloration at the interface whilst the rest of the system appeared unstained. Consistent with this calcium-specific signal, SEM imaging identified densely aggregated granular mineral particles at the same interface. The accompanying EDX spectrum and atomic-percentage analysis revealed pronounced calcium (Ca) and phosphorus (P) peaks, with a Ca/P ratio of 1.73 (Fig. 5F). Finally, calcium measurements significantly increased, indicating maturation of mineralization over time (Fig. 5G). These results strongly support the formation of amorphous calcium phosphate at the mineralization front, marking the onset of the biomineralization process.

## 4 Discussion

Despite three decades of tissue engineering, the field of regenerative dentistry has not yet provided a clinically translatable, bioengineering solution to treat tooth loss. This challenge -at least partially-stems from an incomplete understanding of the complex interaction between DE-DM cells in the tooth constitution process, largely due to the reliance on the traditional *in vitro* approaches and animal models. Herein, we propose a biomimetic tooth-on-a-chip model to serve as a physiologically relevant research platform to facilitate the field of regenerative dentistry. Our results demonstrate that our tooth-on-a-chip model successfully established a 3D DE-DM cell interface, closely mimicking late bell-stage tooth morphology, and events seen in natural early tooth development including DM cell condensation and odontogenic differentiation, DE cell ameloblast differentiation, and mineralization specifically at the DE-DM cell junction.

With respect to technical aspects of our tooth-on-a-chip platform, to date, the very precise soft lithography process has been favored as fabrication method [23]. However, expenses are high due to clean room production, with inherent limited flexibility for design adaptations [24]. In contrast, 3D-printing allows for easy modification [25] and as evident in this study, was particularly advantageous for screening optimal designs to support regenerative research.

In addition to an optimal production method, a second technical aspect was finding the best application-oriented chip design. A horizontal-parallel design, commonly used for on-chip interfaces, enables cells to laterally attach to hydrogel confined between central pillars [26]. The inter-pillar distance and channel width are critical parameters for caging the gel. Reducing the inter-pillar distance (150–200 μm) and widening the central channel (>800 μm) generates a pressure difference (>500Pa) during gel injection, ensuring liquid flow through the central channel without leaking into side channels [27]. However, reducing inter-pillar distance also increases surface tension, risking the entrapment of air bubbles and poor nutrient distribution. Our Design 3 made an efficient compromise, combining pillars of large-enough size, with a step-shaped “dent” in between, to enhance media diffusion while preventing leakage. This study is the first to combine dent-and-pillar structures, to result in an appropriate interface. In the future, this configuration can be efficiently upscaled for high-throughput applications by standardizing the chip design for integration with commercial lab equipment. For example, multi-interface PDMS chips, each containing 4 interfaces arranged at a 9 mm spacing, could be aligned to fit precisely within a 384-well plate, enabling parallelized cell seeding, media exchange, and reagent addition. Especially, recent sequencing studies emphasize precise dynamic control of environmental triggers throughout culture duration. In this case, microfluidic chips hold the great potential by enabling high-throughput screening and precise, independent regulation of culture conditions, such as oxygen tension, growth factors, and inhibitors.

A third and final practical consideration was the choice of an appropriate gel and the resources of cells. Biologically speaking, the ideal scaffold has similar mechanical properties to pulp tissue, while supporting cell survival and differentiation [19,28,29]. And from an engineering perspective, injectability is essential. Our combined experiments identified fibrin as the most promising scaffold. For future personalized medicine approaches, autologous fibrin could even be derived from a patient's own blood. However, in such a scenario also plasmin activity would greatly vary, requiring extensive tailoring per patient [30,31]. Cell source remains a significant challenge in tooth regeneration, primarily due to the absence of DE cells after tooth eruption. Porcine DE cells offer notable advantages as an alternative resource, given their genetic similarity to humans, availability in large quantities compared to rodents, and ease of access from slaughterhouses, thus reducing experimental animal sacrifice. Furthermore, the key epithelial–mesenchymal signaling axes involved in early odontogenesis (e.g., BMP, FGF, WNT, SHH) are highly conserved across mammals, and porcine tooth development is closer to human in tooth size, crown complexity, and developmental tempo than most small-animal models. Previous studies have demonstrated successful interactions between porcine DE and human DM cells, including tooth regeneration experiments in beagle dogs. Clearly identifying and characterizing inductive signals between porcine DE and human DM cells could therefore offer a highly promising and efficient strategy for regenerating human-sized teeth.

When assessing our results, two key factors were essential. First, re-establishing proper DE-DM cell interaction; and second, creating a tooth bud stage-specific tissue culture environment. Substantial literature has demonstrated that DE cell-to-ameloblast differentiation, reciprocal interaction with DM is essential [2]. The DM cell's TGFβ signaling thereby is identified as a key trigger [33]. Therefore, this study first focused on creating a condensed DM cell niche in the chip, prior to introducing DE cells [6]. In support of this approach, Rosowski et al. demonstrated that condensed DM can act as a signaling reservoir to facilitate dental epithelial cell invagination [5]. Among the critical signaling pathways involved in natural tooth development, BMP4 in particular has been demonstrated to play multiple roles in mediating ameloblast differentiation and enamel maturation [[33], [34], [35]]. Exogenous BMP4 has been shown to enhance DE cell proliferation and to promote the differentiation of embryonic stem cells into ameloblasts [[36], [37]]. Based on the observed upregulation of BMP4 in our model, it is reasonable to propose that DE cell differentiation was driven by BMP4 expression in the DM cells, further supporting the tooth-on-a-chip as a reliable model recapitulating the *in vivo* condition.

Second, an appropriate culture environment is crucial for providing the stage-specific signals necessary to replicate natural tooth development. Organoids after *in vivo* transplantation can result in the formation of reasonably structured hard tissues including dentin and enamel [33]. Still such models, fail to achieve mineralization *in vitro*, and thus have little predictive value. Remarkably, our model showed distinct mineral deposition using postnatal cells *in vitro*, a feature rarely reported by previous tooth regeneration models. This outcome could be explained by establishment of the interface morphology, which appears essential for guiding proper cellular interactions and tissue maturation. Even so, fully qualifying the mineral phase remained challenging: although SEM micrographs and EDX indicated amorphous calcium phosphate, spectroscopic/crystallographic confirmation was constrained by the very low mineral content and short working distances, which yielded poor signal-to-noise in Raman/XRD.

While our current results demonstrate clear evidence of early odontogenesis and amelogenesis, future studies should include dynamic temporal assessments of additional markers and epithelial polarity. For example, evaluating markers specific to preameloblasts, early ameloblasts, and secretory ameloblast stages would provide a quantitative map of ameloblast maturation process. For future refinements to the model, suppression of Shh signaling could be a valid approach. Kim et al. demonstrated that such suppression induced ameloblastin expression and enamel crystal alignment [38]. Also, recent advances in omics-based technologies elucidated spatiotemporal single-cell dynamics and regulatory mechanisms driving natural human tooth development [39]. Integration stage-specific signaling pathways into our model could be used to even more effectively replicate the natural developmental processes [32,35,40]. Moreover, incorporating pluripotent stem cell-derived epithelial or mesenchymal cells (e.g., iPSCs) into our model would substantially strengthen its generalizability, potentially allowing personalized screening and broader translational applications in tooth regeneration.

In conclusion, this study introduces a microphysiological tooth-on-a-chip *in vitro* model successfully replicating early-stage DE-DM cell interaction. By leveraging the adaptability and rapid production capabilities of 3D-printing, we created a model that successfully mimicked the physical and biological processes. Our biomimetic tooth-on-a-chip design recapitulated key developmental stages of embryonic tooth development, including DM cell alignment, condensation, odontogenesis, DE cell differentiation into ameloblasts, and DE/DM cell mineralization, all of which effectively captured the dynamics of early tooth development. We anticipate that these models will enhance the fundamental knowledge needed to support future efforts in regenerative dentistry. Furthermore, by integrating the use of patient-derived cells, we anticipate that our tooth-on-a-chip platform will be adopted as a personalized tool for future studies of a variety of developmental dental disorders such as amelogenesis imperfecta and dentinogenesis imperfecta.

## CRediT authorship contribution statement

**C. Huang:** Writing – review & editing, Writing – original draft, Visualization, Validation, Software, Resources, Project administration, Methodology, Investigation, Funding acquisition, Formal analysis, Data curation, Conceptualization. **F. Sanaei:** Writing – review & editing, Visualization, Validation, Software, Methodology, Investigation, Formal analysis, Data curation, Conceptualization. **W. Zhang:** Writing – review & editing, Supervision, Project administration, Methodology, Investigation, Data curation, Conceptualization. **P.C. Yelick:** Writing – review & editing, Supervision, Project administration, Methodology, Investigation, Data curation, Conceptualization. **W. Ji:** Writing – review & editing, Supervision, Project administration, Methodology, Investigation, Conceptualization. **F. Yang:** Writing – review & editing, Supervision, Resources, Project administration, Methodology, Investigation, Formal analysis, Data curation, Conceptualization. **X.F. Walboomers:** Writing – review & editing, Visualization, Validation, Supervision, Software, Resources, Project administration, Methodology, Investigation, Funding acquisition, Formal analysis, Data curation, Conceptualization.

## Funding

The authors disclosed receipt of the following financial support for the research, authorship, and/or publication of this article: This work was supported by grants from the 10.13039/501100004543China Scholarship Council (202106270158) and 10.13039/501100011970International Team for Implantology (1838–2024).

## Declaration of competing interest

The authors declare the following financial interests/personal relationships which may be considered as potential competing interests: X.Frank Walboomers reports financial support was provided by 10.13039/501100016070ITI International Team for Implantology. If there are other authors, they declare that they have no known competing financial interests or personal relationships that could have appeared to influence the work reported in this paper.

## Data Availability

Data will be made available on request.
